# Plant Responses to Heavy Metals: From Deficiency to Excess

**DOI:** 10.3390/ijms26052064

**Published:** 2025-02-27

**Authors:** Ilya V. Seregin, Anna D. Kozhevnikova

**Affiliations:** K.A. Timiryazev Institute of Plant Physiology, Russian Academy of Sciences, Botanicheskaya st., 35, Moscow 127276, Russia; kozhevnikova.anna@ifr.moscow

Some metals and metalloids such as copper (Cu), manganese (Mn), nickel (Ni), zinc (Zn), iron (Fe), boron (B), and molybdenum (Mo) are essential for most plants but toxic when supplied in supraoptimal quantities; meanwhile, the biological role of cadmium (Cd), mercury (Hg), lead (Pb), and arsenic (As), with rare exceptions, is unknown, and they are toxic for plants even at fairly low concentrations. Both metal deficiency and metal excess can drastically affect different physiological processes in plants, leading to significant changes in plant growth and morphogenesis as well as plant productivity. This Special Issue is focused on the mechanisms of metal effects, plant responses to heavy metals, metal tolerance, and metal (hyper)accumulation at the biochemical, physiological, molecular, genetic, and epigenetic levels.

The review by Xu et al. provides a comprehensive overview of the diverse functions of Cu as an essential element in plant metabolism; the symptoms of Cu deficiency and toxicity; Cu dynamics in soil; the mechanisms of its uptake and root-to-shoot translocation; the intracellular transport of Cu and its accumulation in vacuoles, chloroplasts, mitochondria, and endomembranes; Cu efflux from the cell; and the role of transcription factors in maintaining Cu homeostasis.

The review by Geng et al. summarizes recent studies on the molecular mechanisms of the toxicity, uptake, transport, redistribution, and detoxification of As, a widespread metalloid environmental pollutant, in rice. The review provides novel insights into and approaches to preventing and controlling As bioaccumulation in rice plants, especially in the realm of reducing As accumulation in rice grains. An in-depth understanding of the molecular regulatory mechanisms underlying As uptake, transport, and detoxification in rice is of great significance to solving the problem of As bioaccumulation in rice, thereby improving its quality and safety and protecting human health. 

The review by Seregin and Kozhevnikova summarizes the current knowledge on the biosynthesis of a low-molecular-weight N-containing metal-binding ligand nicotianamine (NA), the mechanisms of NA secretion by plant roots, the mechanisms of the intracellular transport of NA and its complexes with metals, and its role in radial and long-distance metal transport in plants. The accumulation of NA in excluders—storing metals primarily in roots—and in hyperaccumulators—accumulating metals mainly in shoots—is compared, and the role of NA in metal tolerance, maintaining metal homeostasis, and hyperaccumulation mechanisms is also discussed. 

Zhang et al. conducted a comparative transcriptome study between the cadmium hyperaccumulator *Sedum plumbizincicola* and the non-hyperaccumulating ecotype (NHE) of *Sedum alfredii* with and without Cd treatment. Their integrated transcriptome analysis reveals many differentially expressed genes involved in heavy metal transport and detoxification that were abundantly expressed in *S. plumbizincicola* and identifies a large number of differentially expressed transcription factor genes, highlighting the complexity of transcriptional regulatory networks. Four transcription factor genes that were highly expressed in the roots of *S. plumbizincicola* were further screened as candidate genes for creating CRISPR/Cas9 knockout mutations. Among these, the *SpARR11* and *SpMYB84* mutant lines exhibited decreased Cd accumulation in their aboveground parts, suggesting that these two transcription factors may play a role in the regulation of Cd hyperaccumulation in *S. plumbizincicola*. 

Zhou et al. studied the effect of the overexpression of *ApHIPP26* from the hyperaccumulator *Arabis paniculata* on Cd tolerance and accumulation in transgenic *Arabidopsis thaliana*. Transgenic *Arabidopsis* overexpressing *ApHIPP26* exhibited higher Cd accumulation in roots and shoots and a significant increase in main root length and fresh weight compared to wild-type plants under Cd stress. The expression of genes related to the uptake and transport of heavy metals, such as *AtNRAMP6* and *AtNRAMP3*, changed in response to Cd. The cadmium-induced activities of superoxide dismutase, peroxidase, catalase, and ascorbate peroxidase were higher in the transgenic lines compared to the wild type. In addition, the gene expression profile analysis showed that *ApHIPP26* improved the tolerance of *Arabidopsis* to Cd by regulating the changes in related genes in the plant hormone signal transduction pathway. Zhou et al. conclude that ApHIPP26 plays an important role in Cd tolerance by alleviating oxidative stress and regulating plant hormones.

Koźmińska et al. investigated how introducing halophilic sulphur-oxidizing bacteria (SOB) *Halothiobacillus halophilus* to the growth substrate affected the physiological and biochemical responses of the halophyte *Tripolium pannonicum* under salt and Cd stress conditions. Bacterial inoculation was effective in mitigating the deleterious effect of Cd on some growth criteria, such as stem length and growth tolerance index. SOB-inoculated plants exhibited a 20% increase in the content of photosynthetic pigments compared to non-inoculated plants. Furthermore, SOB contributed to enhancing Cd tolerance in *T. pannonicum* by increasing the availability of sulphur in leaves, which led to the maintenance of an appropriate level of phenolic compounds, as well as chloride ions. The level of MDA decreased after bacterial inoculation in all experimental variants except when both salt and Cd stress were present. The data from this investigation suggest that inoculating the substrate with SOB has a beneficial effect on the Cd tolerance of *T. pannonicum*.

ATP-binding cassette (ABC) transporters mediate metal transport and are involved in plant tolerance to various types of environmental stress. Huang et al. functionally characterized two half-size ABC transporters (GmABCB48 and GmABCB52) in soybean (*Glycine max*). Both transporters were localized at the plasma membrane. Expression analysis showed that *GmABCB48* and *GmABCB52* were only induced in roots. The overexpression of *GmABCB48* or *GmABCB52* in *Arabidopsis thaliana* reduced aluminium (Al) accumulation in roots and promoted Al tolerance. Transgenic lines expressing *GmABCB48* or *GmABCB52* had lower Al content in root cell walls than wild-type plants under Al stress. The Al content in the cell wall fractions of transgenic lines was significantly lower than that of wild-type plants, which coincided with the changes in pectin and hemicellulose 1 content under Al exposure. Huang et al. concluded that GmABCB48 and GmABCB52 conferred Al tolerance by regulating the metabolism of cell wall polysaccharides to reduce Al accumulation in roots.

Guo et al. conducted a full-length transcriptome sequencing for aluminium (Al)-treated and -untreated *Stylosanthes* spp. root tips and identified three *Snakin/GASA* genes. Through quantitative RT-PCR, they found that only *SgSnakin1* was significantly upregulated by Al treatments. Histochemical localization assays further verified the Al-enhanced expression of *SgSnakin1* in the root tips. The overexpression of *SgSnakin1* conferred Al tolerance to transgenic *Arabidopsis thaliana*, which was reflected by higher relative root growth and cell vitality, as well as lower Al content in the roots of transgenic plants. Additionally, the overexpression of *SgSnakin1* increased the activities of SOD and POD and decreased the levels of O_2_^·−^ and H_2_O_2_ in transgenic *A. thaliana* in response to Al stress. Guo et al. came to the conclusion that SgSnakin1 may promote Al tolerance by enhancing the scavenging of reactive oxygen species through the regulation of antioxidant enzyme activities.

Liu et al. examined the effect of excess Mn on soybeans (*Glycine max*) and observed root growth inhibition; an increase in the activities of peroxidase, superoxide dismutase, ascorbate peroxidase, and catalase, as well as the contents of proline, malondialdehyde, and soluble sugar; and a decrease in the contents of soluble proteins, indolebutyric acid, abscisic acid, indoleacetic acid, and gibberellic acid 3. In addition, the contents of Mn, Fe, sodium (Na), Al, and selenium (Se) in the roots increased significantly, whereas those of magnesium (Mg), Zn, and potassium (K) decreased. RNA sequencing analysis revealed that 572 differentially expressed genes (DEGs) were upregulated and 858 DEGs were downregulated, indicating that soybean roots may initiate complex molecular regulatory mechanisms in response to Mn excess. This work was continued, and the study by Pan et al. revealed that the treatment of soybean plants with excess Mn resulted in the yellowing of old leaves; significant increases in the activities of peroxidase, superoxide dismutase, ascorbate peroxidase, and catalase; and elevated levels of malondialdehyde, proline, indoleacetic acid, and salicylic acid therein. Conversely, the levels of abscisic acid, gibberellin 3, and jasmonic acid significantly decreased. The Mn content in the affected leaves significantly increased, while the levels of Ca, Na, K, and Cu decreased. Transcriptome analysis revealed 2258 differentially expressed genes in the Mn-stressed leaves, 744 of which were upregulated and 1514 were downregulated. These genes included genes associated with ion transporters, hormone synthesis, and various enzymes. The quantitative RT-PCR (qRT-PCR) verification of fifteen genes confirmed altered gene expression in the Mn-stressed leaves. 

Adamczyk-Szabela and Wolf studied Zn and Cu uptake and effects on four popular medicinal herbs: *Ocimum basilicum*, *Borago officinalis*, *Urtica dioica*, and *Mentha piperita*. Plants grown in soil supplemented with Cu exhibited lower Fe content but higher Mn content in roots. The only exception was *B. officinalis*, where Mn content in roots decreased. Both Cu and Zn treatments increased the total content of phenolics, especially in *U. dioica* and *O. basilicum*. Overall, Cu and Zn did not significantly affect the photosynthesis. Herbal extracts from *U. dioica* and *O. basilicum* had unique antioxidant properties and may be good free radical scavengers.

Zhao et al. performed a detailed comparison of transcriptome differences between two rice cultivars differing in Cd translocation from spike-neck to grain: ZZY (*Oryza sativa* L. ssp. *indica* hybrid, high-translocation–high-grain Cd) and SJ18 (*Oryza sativa* L. ssp. *japonica*, low-translocation–low-grain Cd). Compared with ZZY, 22,367 differentially expressed genes (DEGs) were identified in SJ18, including 2941 upregulated and 19,426 downregulated genes. GO analysis enriched 59 downregulated terms, and KEGG enrichment identified 21 significantly downregulated pathways for these DEGs. WGCNA revealed that these DEGs could be clustered into five modules. The hub genes of the yellow module, which were significantly related to SJ18, included OsHMA- and OsActin-related genes, whereas those of the brown module, which were significantly related to ZZY, included MAPK-, CBS-, and glutaredoxin-related genes. Zhao et al. suggest that different mechanisms are involved in the process of spike-neck–grain Cd translocation among varieties. 

Yang et al. conducted a comparative transcriptome analysis to identify differentially expressed genes (DEGs) in the shoots and roots of *Brassica carinata* when exposed to Cd. A total of 631 DEGs were found in the shoots, while 271 DEGs were found in the roots. These selected DEGs also showed differential expression after exposure to Pb, suggesting that *B. carinata* may employ a similar molecular mechanism when tolerating these heavy metals. The functional annotation of the DEGs showed enrichment in the categories of ‘inorganic ion transport and metabolism’ and ‘signal transduction mechanisms’. Additionally, the DEGs involved in ‘tryptophan metabolism’ and ‘zeatin biosynthesis’ pathways were found to be upregulated in both the shoots and roots of *B. carinata*, suggesting that the plant can enhance its tolerance to Cd by promoting the biosynthesis of plant hormones.

The novel data obtained in the works published in this Special Issue contribute to the understanding of the physiological and molecular mechanisms of metal/metalloid transport and the mechanisms of plant adaptation to metal/metalloid excess and deficiency in the environment. The elucidation of these mechanisms is of significant practical importance since it underlies the development of approaches used in the technologies of phytoremediation, phytomining, biofortification, and sustainable and environmentally friendly agriculture.

